# Detection of Neovascularization Based on Fractal and Texture Analysis with Interaction Effects in Diabetic Retinopathy

**DOI:** 10.1371/journal.pone.0075699

**Published:** 2013-12-16

**Authors:** Jack Lee, Benny Chung Ying Zee, Qing Li

**Affiliations:** Division of Biostatistics, Jockey Club School of Public Health and Primary Care, The Chinese University of Hong Kong, Shatin, New Territories, Hong Kong; Medical University of Graz, Austria

## Abstract

Diabetic retinopathy is a major cause of blindness. Proliferative diabetic retinopathy is a result of severe vascular complication and is visible as neovascularization of the retina. Automatic detection of such new vessels would be useful for the severity grading of diabetic retinopathy, and it is an important part of screening process to identify those who may require immediate treatment for their diabetic retinopathy. We proposed a novel new vessels detection method including statistical texture analysis (STA), high order spectrum analysis (HOS), fractal analysis (FA), and most importantly we have shown that by incorporating their associated interactions the accuracy of new vessels detection can be greatly improved. To assess its performance, the sensitivity, specificity and accuracy (AUC) are obtained. They are 96.3%, 99.1% and 98.5% (99.3%), respectively. It is found that the proposed method can improve the accuracy of new vessels detection significantly over previous methods. The algorithm can be automated and is valuable to detect relatively severe cases of diabetic retinopathy among diabetes patients.

## Introduction

The retinal vasculature is an observable circulatory system in the eye [1]–[4] without using any invasive procedure, which provides useful information about the microcirculation in the body. Neovascularization is a pathognosmatic sign of proliferative diabetic retinopathy. Effective computer aids could improve sensitivity and consistency of neovascularization detection during regular follow-up visits or telemedicine consultations. More reliable detection would decrease the possibility of patients missing timely and effective laser treatment. Unlike microaneurysms, neovascularization shape and size varies, presenting extra challenges and requirements for the development of automated detection systems.

**Figure 1:**
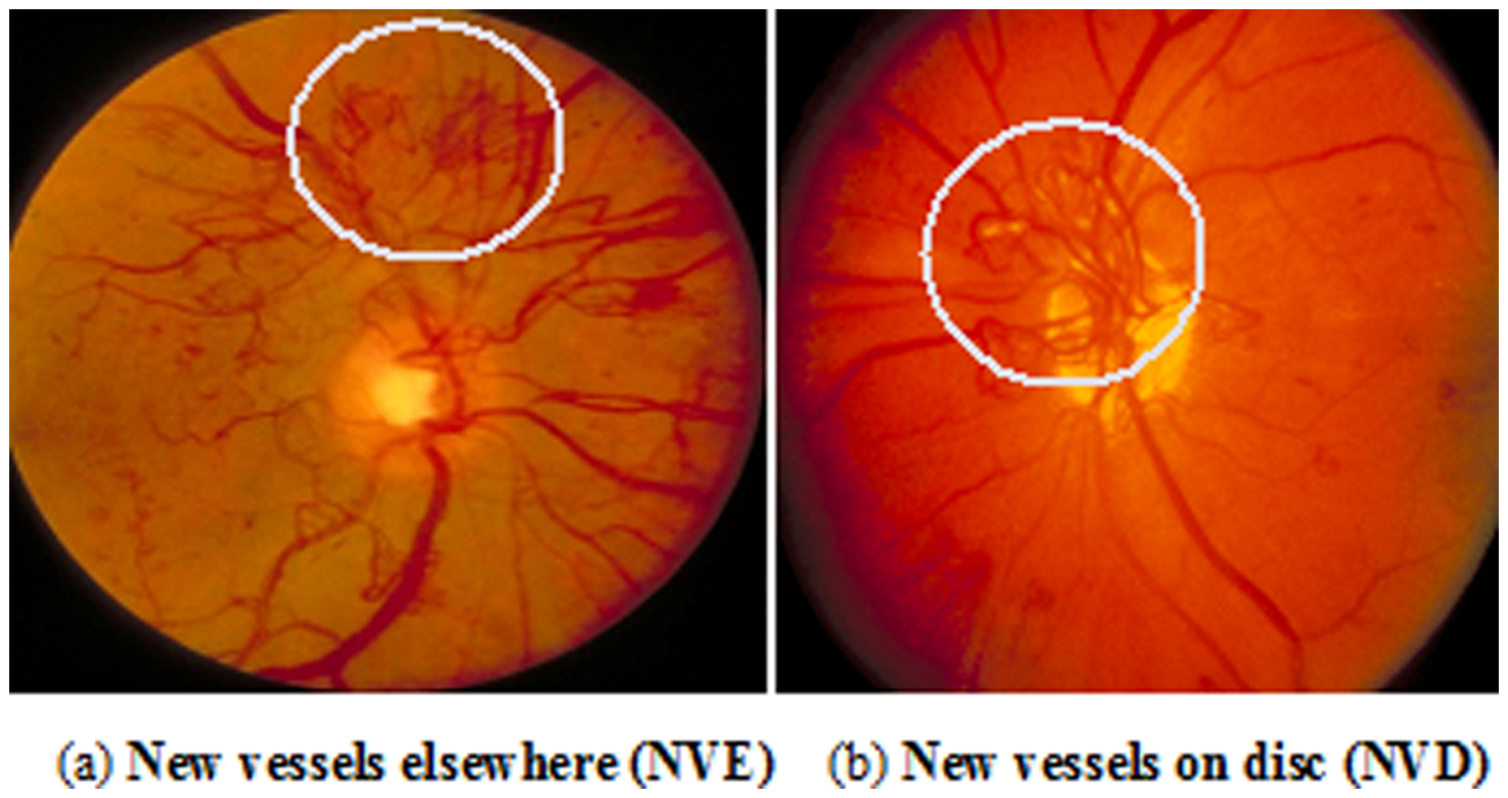
New vessels examples: (a) New vessels elsewhere (NVE). (b) New vessels on disc (NVD).

Neovascularization is one of the hallmarks of proliferative diabetic retinopathy. It is a process whereby the vasogenic factors respond to hypoxia leading to new vessels development. The new vessels are defective and leak fluid (edema/true exudates) and red cells (hemorrhages). This in turn stimulates connective tissue growth. When neovascularization is present, either in the form of neovascularization on the disc (NVD) or neovascularization elsewhere (NVE), it prompts a mandatory referral to a retinal specialist or ophthalmologist. An example of NVD and NVE is presented in Figure 1.

There are many studies provided automatic detection of retinal abnormalities for microaneurysms, hemorrhages, hard exudates and cotton wool spot. However, research on the detection of neovascularisation is relatively rare, mainly due to the fact that it is difficult to distinguish normal blood vessels from new vessels. Intrusive methods using radio-opaque contrast agent injected into the blood stream and the diagnosis is done manually by ophthalmologist based on the angiograph [5]. Even though angiography-based methods could produce detailed retinal image, they are usually not preferred especially for early stage or regular diagnosis because of the intrusive nature of this method.

Numerous studies on new vessels detection using the retinal image based noninvasive approaches have been proposed. For example, Saranya et al. segmented vessels using FCM technique, the features included Gradient, Gradient variation, Gray level coefficient of variation, moment invariants-based features and tortuosity, which mainly rely on shape, contrast, and brightness on segmented vessels [6]. Unfortunately this approach is not fully automatic. Goatman K.A. et al. provided a method for detecting standard screening photographs which show new vessels on the optic disc. Of all the 15 features of proliferative retinopathy, new vessels at the optic disc can be detected [7]. This approach is not useful for new vessels outside of the optic disc area (NVE). Hassan et al. presented a holistic non-intrusive approach using combination of techniques such as compactness classifier, morphological operator, Gaussian, and thresholding [8]. A Region-level based technique where number of vessels and areas of vessels were used to classify new vessels. This is also not fully automatic. Other studies included S. Nithyaa and S. Karthikeyen [9], they segmented vessels by using watershed transform. Six statistical features including skewness, kurtosis, entropy, energy, correlation and RMS (root mean square) were used. This approach could only detect new vessels on the optic disc. C. Agurto et al. applied AM-FM Representation and Granulometry to extract features (AM-FM types of features) and vessel segmentation on ROI by adaptive vessel segmentation (consecutive level for enhancement). This approach only detected new vessels on the optic disc (NVD) and the number of samples is relatively small [10]. Finally M.U. Akram et al. applied segmented vessels by using a recursive supervised multilayered thresholding-based method. This approach used features that mainly based on different vascular properties such as vessel density, edge magnitude etc. They used features such as energy, mean gradient, standard deviation gradient, mean intensity and intensity variation [11]. This approach provided a higher accuracy of detecting new vessels but required a large sample size comparing to previous methods. From the above, we can see that previous methods either have limitation for the detection of neovascularization within NVD, or that they are not fully automatic. Most importantly, all of the previous methods did not consider interaction effects among the selected features on neovascularization while the complexity of neovascularization requires us to identify them more accurately by considering the interaction effects from their characteristics. In this study, we present a novel approach of detecting neovascularization using an integrated technique of statistical texture analysis (STA), high order spectrum analysis (HOS) and fractal analysis (FA). Features selected from this approach, incorporating their associated interactions are also considered.

## Methods

### 2.1 Retinal imaging and acquisition

In this study, the fundus images from two different public databases were used. They are MESSIDOR database [12] and DIARETDB0 database [13]. Both of them have been established to facilitate studies on computer-assisted diagnoses of diabetic retinopathy. For MESSIDOR database, there are 1200 eye fundus color numerical images of the posterior pole acquired by 3 ophthalmologic departments using a color video 3CCD camera on a Topcon TRC NW6 non-mydriatic retinograph with a 45 degree field of view. The images were captured using 8 bits per color plane at 1440*960, 2240*1488 or 2304*1536 pixels. Among these images, 800 images were acquired with pupil dilation (one drop of Tropicamide at 0.5%) and 400 without dilation. All images are in TIFF format. For DIARETDB0 database, it consists of 130 color fundus images of which 20 are normal and 110 contain signs of the diabetic retinopathy (hard exudates, soft exudates, micronaneuyrysms, hemorrhages and neovascularization). Images were captured with a 50 degree field-of-view digital fundus camera with unknown camera settings.

**Figure 2:**
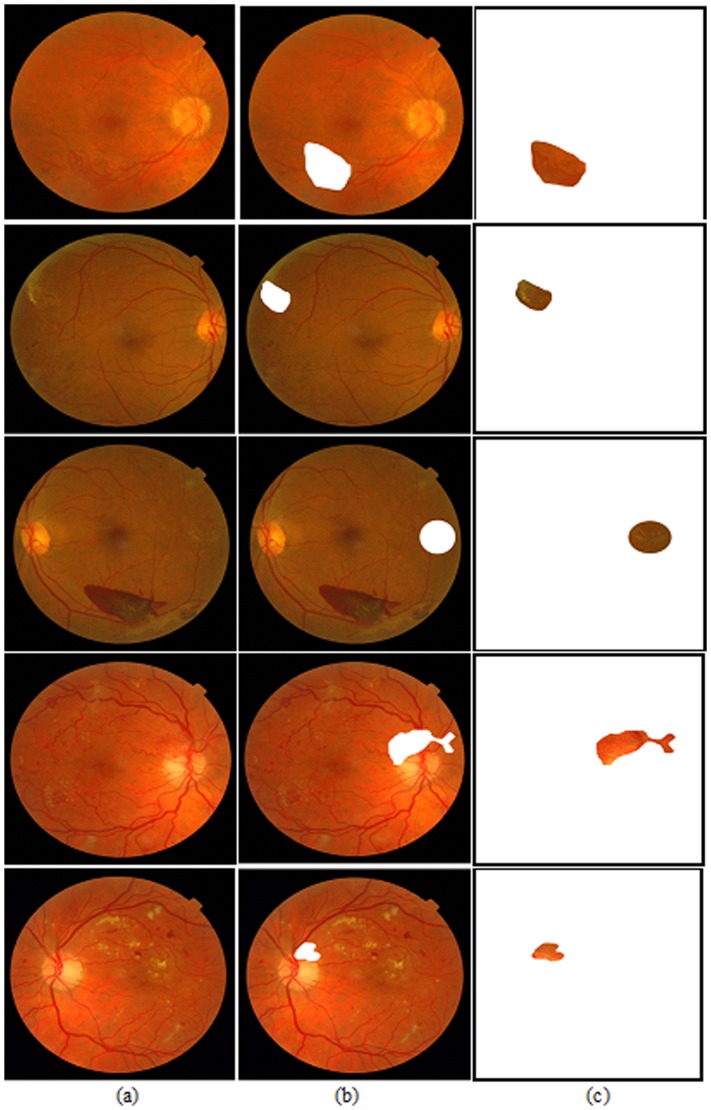
(a) the first column is original images; (b) the 2^nd^ column is original images with discarded regions of NVE/NVD and (c) the 3^rd^ column is the discarded regions of NVE/NVD.

All the images in this study were selected from the benchmark retinal databases that are available online. Thus, detail of selection process or enrollment procedures of the patients is available through their respective websites. Certain criteria were set to choose suitable neovascularization image. From these two online databases, only 137 were used in our study because we have excluded the images that are not clear or with low resolution, and the ground truth not related to diabetic disease are discarded. Thus only 7 neovascularization cases were selected from MESSIDOR database,, and only 20 neovascularization cases were selected from the DIARETDB0 database. Therefore, a total of 27 images were chosen with indicated ground truth and have neovascularization. For the 110 controls images without neovascularization, all of them were selected from the DIARETB0 database. Figure 2 shows a sample of the 5 images with NVE or NVD cases we have used in this study as test cases. Notice that these five test cases all have neovascularization rated by an eye-expert in MESSIDOR database. First three cases are neovascularization with the NVE. Last two cases are neovascularization with the NVD (or partially NVD).

### 2.2. Methodology

**Figure 3:**
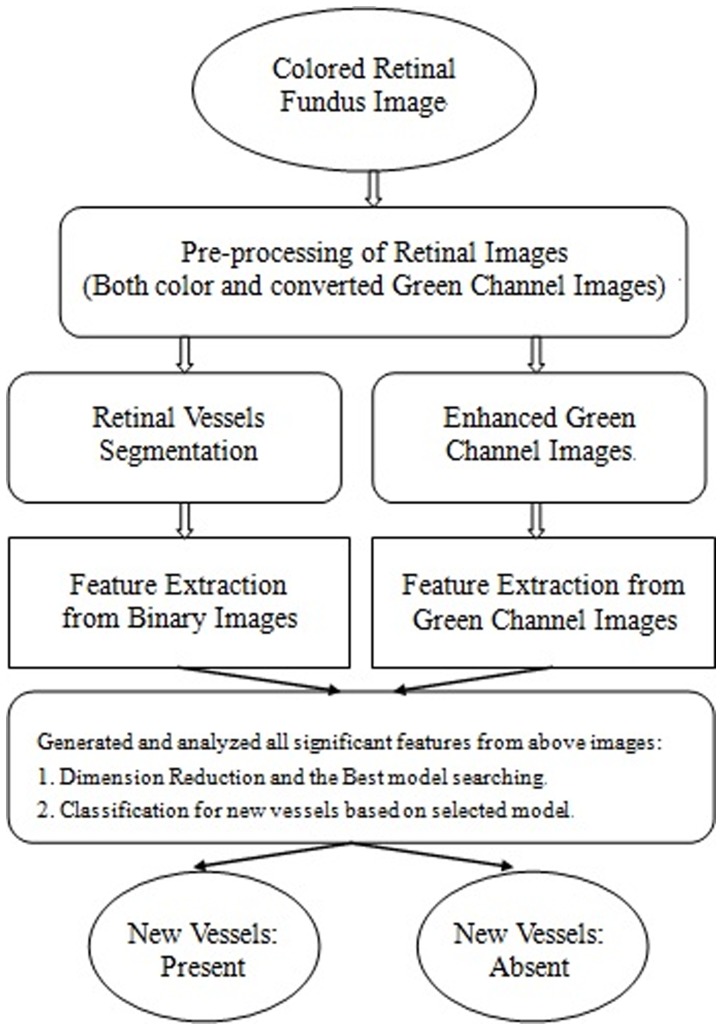
The baseline structure of the new vessel-detection in retinal image analysis.

We mainly focus on the extraction of all possible significant risk factors that highly associated with new vessels, especially concentrated on the vessel related characteristics. We selected the green channel for all of our operations since retinal images are almost always saturated in the red channel and have very low contrast in the blue channel. The whole scheme of system is provided in Fig. 3.

#### 2.2.1 Preprocessing

The purpose of preprocessing and contrast enhancement is to remove any artifacts that occur during retinal image acquisition process. We applied the following procedures to preprocess the raw images.

1. Convert the colored (RGB) image to green channel image and process the contrast and enhancement. Perhaps one of the most basic enhancement techniques is the contrast stretch. We applied the **decorrelation stretching** method for the enhancement works by increasing the differences in hue [14], this technique gives better results for microvasculature in retinal image (provide more vessels' information).
2. We then applied the hybrid median filtering method to separate vessels from others. This approach is the windowed filter of nonlinear class that can easily remove impulse noise while preserving edges (during the vessel segmentation). We first generates ranking in multiple sub-neighborhoods for each pixel and then takes the median values from those rankings and performs a final ranking to select the final result. This procedure better preserves fine details while removing the noise. More details can be seen in [15].

**Figure 4:**
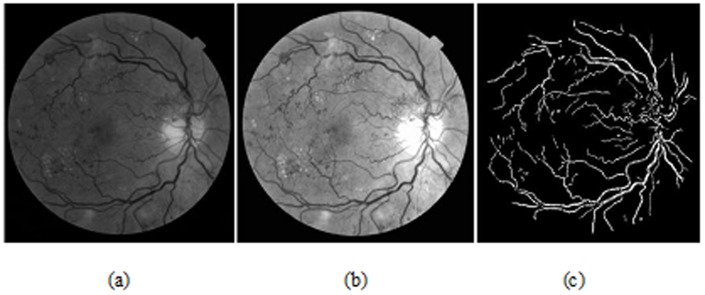
Retinal image after preprocessing; (a) original green channel image; (b) enhanced/preprocessed image; (c) vessels extracted image.

In image analysis, Non Uniform Illumination in an image often leads to diminished structures and inhomogeneous intensities due to different texture of the object surface and shadows cast from different light source directions. It is one of the most important factors affecting the appearance of an image, more adverse in case of biological images. For the correction of non uniform illumination, we apply the morphological operations. The basic idea is as follow: display the background approximation as a surface first, and then under the assumption that the vessels maintain predominantly in local linear orientation we select a line structure to generate a morphological filter for vessel segmentation [16]. This approach was used to solve the problem by estimating background approximation as a surface in order to extract the non-uniform background from the image, and then construct the new image by subtracting this estimated background from the original image. Results reveal that various particles present in the image with exact boundaries along with the removal of non-uniform illumination at the background. Basic morphological operators are erosion, dilation, opening, closing as defined in [16]. The top-hat, bottom-hat, and morphological enhancement operators are written as
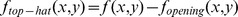


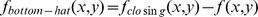


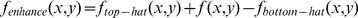
(1)Because blood vessels are darker than the background in retinal image, a bottom-hat operator and enhancement operator are used to generate the feature response images. After this, we applied the dual-tree complex wavelet (CWT) on the above enhanced image since it is a valuable enhancement of the traditional real wavelet transform that is nearly shift invariant and, in higher dimensions (is oriented in 2D here), directionally selective [17]. At this point, the image denoising task was completed and we have eliminated most of the small or rough artifacts that may not be detected and removed by the above two approaches. This is especially beneficial for the vessels extraction. Other alternative way is to apply the matched filters approach on this enhanced image. An example of the above preprocessed result is presented in Fig. 4 (sample images from a public database of MESSIDOR).

In the next section we will discuss features extraction and formulation. There are three approaches for the texture analysis: 1) Statistical texture analysis involving gray level co-occurrence matrix (GLCM) and run-length matrix, 2) HOS analysis and 3) fractal analysis to generate useful features from the enhanced image. There are a total of 75 features generated initially. It includes 50 features coming from HOS analysis and 17 features generated from statistical texture analysis, and 8 features (three from mono-spectrum and five from multi-spectrum) from fractal analysis.

#### 2.2.2 Features extraction and formulation - Statistical based texture analysis

All related texture features from the statistical-based texture analysis were generated from each retinal image that derived from gray level co-occurrence matrix (GLCM) and run-length matrix [18]–[26]. For an image of size M×N, the gray level co-occurrence matrix (GLCM) is defined as in [20]

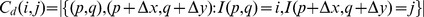
(2)Where (*p, q*), (*p+*Δ*x, q+*Δ*y*)∈*M×N, d* = (Δ*x*, Δ*y*) and |⋅| denotes the cardinality of a set *I(p,q)* indicates the gray level of a pixel at *(p,q)* in an image. Thus given a gray level *i* in an image, the probability that the gray level of a pixel at a (Δ*x*, Δ*y*) distance away is *j* is
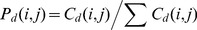
(3)From each co-occurrence matrix we computed the following features:
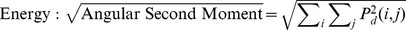


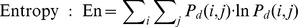


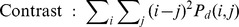


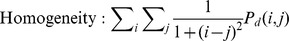
Energy measures the textural uniformity of the image, i.e., the repetition of pixel pairs. It is the measurement of the denseness or order in the image. Entropy measures disorder or randomness of the image and it is an indication of the complexity within an image, thus, more complex images have higher entropy values. Contrast is a measure of the presence of local variations (or differences in the GLCM) in the image, and higher contrast values indicate large local variations. Homogeneity (also called an inverse difference moment) is inversely proportional to the contrast at constant energy. Similarly at constant contrast, homogeneity is inversely proportional to energy [27]. It measures how close the distribution of elements in the GLCM is to the diagonal of GLCM.

Other measurements such as Moments 1–4 are defined as:
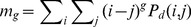
(4)where *g* is the integer power exponent that defines the moment order. Moments are the statistical expectation of certain power functions of a random variable and are characterized as follows [20]: moment 1 is the mean which is the average of pixel values in an image [21]; moment 2 is the standard deviation; moment 3 measures the degree of asymmetry in the distribution; and moment 4 measures the relative peakedness or flatness of a distribution and is also known as kurtosis [22].

Other similar approach can also be used: having the GLCM normalized, we can then derive eight second order statistic features which are also known as haralick features [23] for each image, which are: contrast, correlation, energy, entropy, homogeneity, dissimilarity, inverse difference momentum, maximum probability. In addition to these features, we also applied correlation, dissimilarity, inverse difference momentum and maximum probability, which is different from above mentioned features.

The gray level run-length matrix (RLM) is defined as the numbers of runs with pixels of gray level *i* and run length *j* for a given direction [24]. RLMs was generated for each sample image segment having directions (0°,45°,90° & 135°), then the following five statistical features were derived: short run emphasis, long run emphasis, gray level non-uniformity, run length non-uniformity and run percentage [26]. Basically it allows extraction of higher order statistical texture features.

#### 2.2.3 Features extraction and formulation - High order Spectra analysis (HOS)

**Figure 5:**
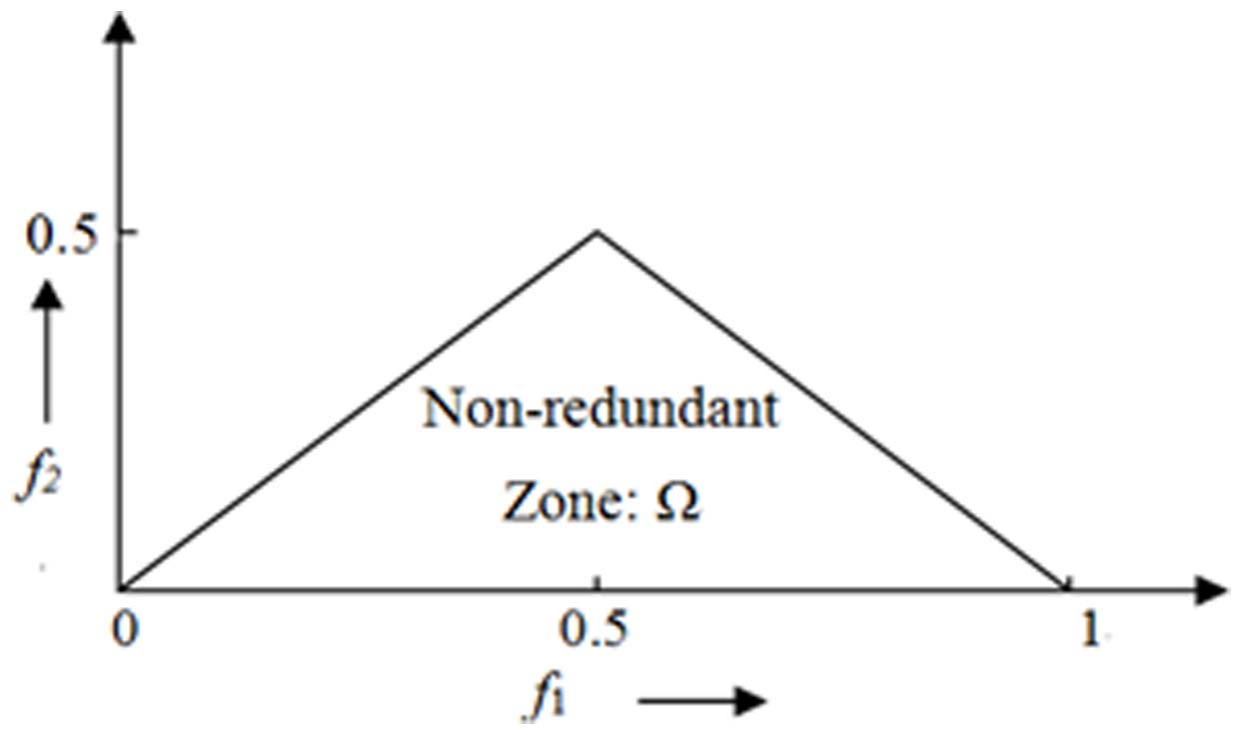
Non-redundant region (Ω) of computation of the bispectrum for real signals. Parameters are calculated from this region.

Higher order spectra (HOS) are known to have the ability to detect non-linearity and deviations from Gaussian assumption. Motivated by these, a set of HOS based parameters were proposed as features to differentiate the new vessels from normal vessels. HOS are spectral representations of higher moments and they are derived from the averaged Fourier spectrum signal. The bispectrum *B*(*f_1_, f_2_*), of a signal is the Fourier transform (FT) of the third order correlation of the signal. It is given by
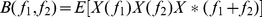
(5)where *X( f)* is the FT of the signal *x(nT)*, * represents complex conjugation and *E[.]* stands for the expectation operation. It retains Fourier phase information. The frequency f may be normalized by the Nyquist frequency to be between 0 and 1. The bispectrum, given by equation (5), is a complex-valued function of two frequencies. The bispectrum which is the product of three Fourier coefficients exhibits symmetry and need only be computed in a non-redundant region. Assuming that there is no bispectral aliasing, the bispectrum of a real-valued signal is uniquely defined with the triangle *0≤f_2_≤f_1_≤f_1_+f_2_≤1*.This is termed as Ω, the principal domain or the non-redundant region (See the triangle region in Fig. 5).

Briefly, HOS based and spectral based features are: Mean of spectral magnitude for HOS [28]:
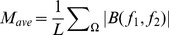
(6)where *B*(* f_1_*, *f_2_*) is the bispectrum of the signal.

Other features such as Entropy 1, 2 and 3 are defined as:
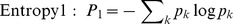


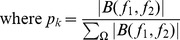


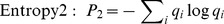


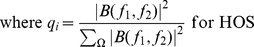


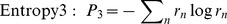


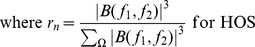
And the feature of bispectrum phase entropy (EntPh):
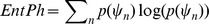
where
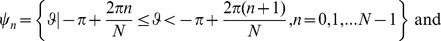


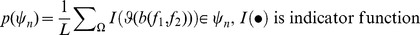

*φ:* Bispectrum phase angle

*L*: Number of points within the samples

We used each of these bispectral invariant features for every 18° from 0° to 180°. Therefore we obtained total 50 features of HOS. Next we will discuss the model-based methods such as fractal model approach. The fractal model is useful for modeling certain natural textures that have a statistical quality of roughness at different scales, and also for texture analysis and discrimination.

#### 2.2.4 Features extraction and formulation - Fractal analysis (FA)

Fractal model (analysis) may be considered as the model-based texture analysis. We can classify it into mono-fractal and multi-fractal. Fractals are geometric objects whose increasing details under magnification resemble exactly or statistically the whole object (self-similarity). Such fractal objects are not easily “measurable” in classic geometric terms because some of their physical characteristics (length, mass, area, volume, and so on) are largely dependent on the magnification used when they are measured. This means that the surface of the fractal object is often complex and any patterns presented on the surface may be combined with different physical characteristics. The diversified complexity of fractal may be described with the concept of fractal dimension [29], which may easily describe the incompleteness or fragmentation of an entirety. Moreover, recent studies show that such surface complexity of image may be described not only by its fractal dimension but also its multifractal spectra, which is mathematical description of a surface that can accurately reflect its features, and is compatible with the various theoretical models that related to surface structures. Thus we can apply fractal analysis to determine the surface complexity which is measured on the gray scale, and by using multifractal spectra one can obtain more detailed information than is possible with the fractal dimension alone. Recently this kind of technique is widely used in retinal vessels analysis.

Recalling fractals are characterized by scale invariance and it always shows a similar degree of covering space (shape). The way in which scale invariance appears is characteristic for the considered structure and can be expressed by a single value, the fractal dimension (FD). FD is a non-integer value which reflects the structure's “convoluteness”. An excellent introduction of fractal analysis can be found elsewhere [30]–[31]. It has also been speculated that fractal analysis could be helpful in diagnosing diabetic retinopathy [32].

Since the retinal vasculature is a fractal that follows the theory of fractal geometry [30], [32]–[33], the fractal dimension is expected to be a natural measure of new vessel formation. It has also been shown that the new vessel formation changes the fractal dimension of the vessel pattern. The fractal dimension exhibits a high degree of sensitivity with respect to new vessel formation, while the image preprocessing with respect to the representation of the individual vessel thickness did not affect the results. The fractal dimension appears to be the “natural” measure for proliferative changes and could be used for automated detection of proliferative diabetic retinopathy in the future [34].

Measuring fractal dimension has previously been attempted to quantify small changes to the human retinal vasculature, not immediately apparent by human observation, and act as an early marker of disease [35]–[37]. Mono-fractal analysis is an indicator of vascular change, it has achieved limited success as retinal vessels may have different characteristics found on different location or with different scale of measurement. Greater success has been reported by considering the retinal vascular pattern to be multi-fractal, characterized by a hierarchy of exponents rather than a single fractal dimension [38].

We first applied box-counting algorithm (with Hausdorff dimension) approach to calculate fractal dimension in binary type of image (segmentation of vessels) and then the Fourier fractal dimension (FFD) approach, which have been proposed by [35]–[37]. FFD has been used to quantify the grayscale images projected on to 3-D fractal surface [38]. The advantage of FFD is that it computes the fractal dimension of gray scale images, and eliminates the need for image segmentation [29]. It has also been found to be relatively insensitive to noise and it is believed to work effectively with data having low signal-to-noise ratio [38]–[39]. We adapted the similar FFD approach proposed by Azemin M. and et al. [37]. The parameters such as slope and intercept were generated based on this approach. Finally we applied the multifractal spectra since it can describe the evolution of the probability distribution of fractal structures [35], [38]–[40]. Instead of using simple fractals (or monofractals), multifractals are characterized by a hierarchy of exponents, rather than a single fractal dimension. In this paper the box-counting method was used to characterize multiple spectra. [38].

Multifractals main parameter is Hólder's exponent [41]–[42].
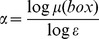
Where *μ*(*box*) represents dimension of box and ε dimension of longitude of box.

Notes: Local and global information from the spectrum are used for segmentation, noise deletion and edge detection at picture points. Segmentation is an important step for description of the basic individual process. One of the commonly used approaches is filtering, but this approach has a main disadvantage on the lost of precision due to preliminary filtering.

Alternative approach is observing of the image as measure of the fix resolution. Irregularities of this measure can be examined with the help of multifractal analysis. The general principle is as follow:

- First, different measures and capacities are defined from image with a gray level.
- After that corresponding multifractal spectrum has to be calculated, enabling local (using α) and global (using f(α)) information. No hypothesis about the signal regularity was used.

Importance and advantage of fractal and multifractal analysis (MFA), compared to “classical” signal analysis is one way of handling of irregularities. MFA tries to extract the information directly from the singularity, whereby “classical” approach often observes LF (low-pass filter) filtered versions, with different filtering depths for irregularities observing and noise repressing. Based on specific values of α and f(α), a homogeneity point can be isolated in original signal. By image pixel extraction, which satisfy chosen value of the parameter α or spectrum f(α), it is possible to extract by any of the known methods. Additional advantage is that such segmentation causes no degradation of the starting image: all pixels interrelations stay unchanged and therefore image details are being completely kept. In order to describe fast changing signals (small area) of retinal image and to express variability, we need to examine fractal characteristic. Usage of classic statistical methods in such case (mean values) can cause valuation errors. Significant singularities are indicated by multifractality of the process.

It is also concluded that the fractal dimension is a descriptor of early changes of pathological vessels changes, which introduce further understanding of the mechanisms of complex changes. Fractals are very important in both medicine and pathology. In our study, five parameters were generated from multifractal analysis: three from local (α) and two from global (f(α)).

Based on the above three approaches, the features set formulated from the texture analysis is given as *I_t_* = {*τ_1_, τ_2_, …, τ_n_*}; where n = 75 is the number of features set for each image.

### 2.3. Dimension Reduction and Best Model Searching

Since there are a total of 75 related factors for the analysis, but the number of samples is limited thus we need to reduce the dimension (filtering out some not-as-useful factors). This will help us find a more suitable diagnostic model in order to detect new vessels with higher efficiency. The following are the two major steps.

#### 2.3.1 Dimension reduction

We first reduce the dimension since some of the features may be correlated or redundant in the model. A penalized logistic regression method (Pelora) [43] was used to cluster the datasets from the above procedures combining all 75 parameters (factors). This is a supervised clustering algorithm that has been used with external information about response variables for clustering genes in genetic study. This algorithm is mainly based on penalize logistic regression analysis and it combines feature selection, supervision, feature clustering and sample classification in a single step. This approach has an advantage of dealing with unbalanced problem (outcomes) since penalized likelihood is a general approach to reducing small-sample bias in maximum likelihood estimation [44]. On the other hands, this approach is different from the classical logistic regression in that it groups features into clusters instead of extracting individual features. This method was used for features selection with the purpose of dimension reduction generated from retinal images and then classified the variables (potential factors) obtained. This approach will preserve the property of the classified (identified) groups with complex interactions.

#### 2.3.2 Model selection with multimodel inference

After the dimensions have been reduced, we applied the method of automated model selection and model-averaging that provides a wrapper for GLM and similar functions, automatically generated all possible models with the specified response of exudates(s) and explanatory variables, and determine the best models with a defined criterion (e.g. AIC or AICc). The best model is mainly based on the bias-variance trade-off.

Statistical models with probability density functions were used, where we can maximize the likelihood
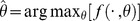
by minimize the Kullback-Leibler distance from the true model to the approximating model, i.e.,
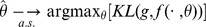
Here *KL* distance is one way to measure the distance between two densities:
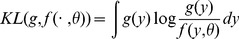
(7)
*g*( *y*) for the discrepancy is weighted by the probability observing the data y, and *g( y)/f( y,θ)* is the ratio that measures the discrepancy between the two models for data y. AIC selects the model closest to the true model on average. Since the true distribution of *g* is unknown, so we replace it with the empirical (observed) distribution. Thus the estimation of *KL* distance is introduced and a bias will also be given. To determine the expected value of this bias, the Takeuchi Information Criterion (TIC) was used. Finally under the assumption of correct approximate model, we derived AIC and related criteria.

In order to deal with the model selection uncertainty and usual sampling uncertainty, we applied AIC to select the model closest to the true model on average (Atypical datasets have lower influence than typical ones). To do this, we first apply bootstrap technique to resample the data based on our raw data and then produce the distribution of best models. For instance, after boostrap data we apply the smoothed model weights and finally determine: 1) the value of the parameter on average (also its variance) for solving the model selection uncertainty problem and, 2) the standard error for a given model that follows a 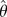 distribution (conditional variance of the estimator), this is to deal with usual sampling uncertainty. Thus we can combine the above two variances to form the overall variance for the estimator 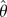. Such approach reduces the spurious estimates (false positive) problem, which standard model selection techniques often encountered (see Freedman's paradox [45]).

We then applied GLMULTI [46], an R package for automated model selection and multi-model inference with GLM and related functions. The basic idea of this approach is that from a list of explanatory variables, GLMULTI builds all possible models involving these variables and, optionally, included their pair-wise interactions (for the computing of simplicity and easy interpretation reasons we will not consider higher order interaction in this paper). Restrictions can be specified for candidate models, by excluding specific terms, enforcing marginality, or controlling model complexity. Detailed information is referred to [46],[47]–[49].

## Results

To evaluate the result for detecting new vessels, we apply a logistic regression classifier to classify new vessels. The receiver operating curve (ROC) was used to assess the performance of neovascularization detection and also the area under curve (with their standard deviations), sensitivity and specificity are obtained to evaluate the classification of neovascularization detection. The result is image based.

In order to validate the importance of inclusion of the interaction effects in the classifications, we used two approaches: 1) We used the features extracted from commonly used texture analysis (i.e., HOS and STA approaches since their features are similar to most of the previous studies mentioned in section one). For this approach, we compare the results with and without interaction effects between extracted features. 2) We used the features extracted from HOS, STA and also include the features from FA, again we also compared the results with and without interaction effects. This analysis demonstrates the importance of fractal analysis features and also the importance of the interaction effects in the course of identification of neovascularization.

As we know, neovascularization occurs due to deteriorated diabetic retinopathy conditions. It is a critical stage for intervention, but the probability of having neovascularization is low from the population of diabetic cases. As mentioned in previous section, we applied fundus images from two different public databases (7 images with neovascularization that selected from MESSIDOR [12] and all 130 images from DIARETDB0 [13]). Thus there are total 137 cases involving 110 without neovascularization (control) and 27 with neovascularization.

The software used to quantify the retina characteristics including SPSS 16.0, R2.13 and Matlab R2011b. Our automatic neovascularization algorithm is running on the platform of Matlab. (Notes: The original retina images from MESSIDOR were stored originally as JPEG format (convert from TIFF format: for the concerning of compression setting to store and fast computing time) with 1440×960, 2240×1488, and 2304×1536 pixels. And the retina images from DIARETDB0 database were originally stored as PNG format with 1500×1152 pixels.) The analyzed results are presented in Table 1.

**Table 1:** Results of neovascularization detection on retinal images.

	Texture Analysis			
Results	(Higher Order Spectra (HOS) and Statistical Texture Analysis (STA))			
	Without Fractal Analysis		With Fractal analysis	
	Without interaction	With interaction	Without interaction	With interaction
**Sensitivity**	74.1%	77.8%	81.2%	96.3%
**Specificity**	98.2%	98.2%	98.2%	99.1%
**Accuracy**	93.4%	94.2%	94.9%	98.5%
**AUC**	96% (SE: 0.21)	97% (SE: 0.16)	96.7% (SE: 0.02)	99.3% (SE: 0.005)

The results show that the use of high order spectra (HOS) and statistical texture analysis (STA) already achieve 74.1% sensitivity and 98.2% specificity with AUC of the receiver operating curve of 96%. Incorporating fractal analysis would significantly increase the sensitivity to 81.2% instead. However, on top of the fractal analysis, the incorporation of interaction effects in the model would further increase significantly the sensitivity to 96.3% and an increase of specificity to 99.1%, with AUC of 99.3%. This result clearly illustrated the importance of interaction effect in the detection of neovascularization. With this result we also carried out a comparison with other existing methods (Table 2). As we have discussed in the introduction, all the currents method are either not fully automatic or suffered from the limitation that they could only detect new vessels inside the optic disc. Our result has achieved the highest sensitivity and specificity for both NVD and NVE in an automatic fashion.

**Table 2:** Comparison of neovascularization detection methods.

Method/Reference	Validation (%)			
(Different dataset)	Sensitivity	Specificity	Accuracy	AUC
**Our propose method**	96.3	99.1	98.5	99.3
Saranya and et. al. [6]	96.25	89.65	96.63	------
Goatman and et al. [7]	82.4	85.9	------	91.1
Syafinah and et al. [8]	63.9	89.4	------	------
Nithyaa and Karthikeyen [9]	92	------	------	94.7
Agurto and et al. [10]	93(96)	60(83)	------	85(94)
Akram and et. al. [11]	96.35	98.93	98.37	

## Discussion

Detection of neovascularization is a difficult and challenging task for retinal abnormality detection since the newly formed blood vessel is tiny and not clearly visible. Our proposed method for automated neovascularization detection is effective for images with different pixel resolution and different type of database (from different fundus camera). The method also achieves fast neovascularization diagnosis by integrating all three different approaches: statistical-based texture analysis, higher order spectra analysis and fractal analysis which included both mono-fractal analysis and multi-fractal (spectrum) analysis. Such integration involved interactive effect between these extracted parameters (useful features) that associated with neovascularization. Its intension is to support the endocrinologist in diabetic retinopathy screening process. The algorithm is developed to process each image automatically not only for detection of NVD but also NVE.

In summary, we have developed methods to improve the accuracy of diagnosis for neovascularization significantly over previous methods. It was determined that application of statistical texture analysis, high order spectral analysis, and fractal analysis together with the incorporation of selected interaction effects, the accuracy of new vessels detection would be significantly improved. The automated approach is valuable for future study of diabetic retinopathy grading system.
